# A specific olfactory bulb interneuron subtype Tpbg/5T4 generated at embryonic and neonatal stages

**DOI:** 10.3389/fncir.2024.1427378

**Published:** 2024-06-12

**Authors:** Akio Tsuboi

**Affiliations:** Graduate School of Pharmaceutical Sciences, Osaka University, Toyonaka, Japan

**Keywords:** Tpbg/5T4, olfactory bulb, granule cells, activity-dependent development, fate map

## Abstract

Various mammals have shown that sensory stimulation plays a crucial role in regulating the development of diverse structures, such as the olfactory bulb (OB), cerebral cortex, hippocampus, and retina. In the OB, the dendritic development of excitatory projection neurons like mitral/tufted cells is influenced by olfactory experiences. Odor stimulation is also essential for the dendritic development of inhibitory OB interneurons, such as granule and periglomerular cells, which are continuously produced in the ventricular-subventricular zone throughout life. Based on the morphological and molecular features, OB interneurons are classified into several subtypes. The role for each interneuron subtype in the control of olfactory behavior remains poorly understood due to lack of each specific marker. Among the several OB interneuron subtypes, a specific granule cell subtype, which expresses the oncofetal trophoblast glycoprotein (Tpbg or 5T4) gene, has been reported to be required for odor detection and discrimination behavior. This review will primarily focus on elucidating the contribution of different granule cell subtypes, including the Tpbg/5T4 subtype, to olfactory processing and behavior during the embryonic and adult stages.

## 1 Introduction

**Figure 1:**
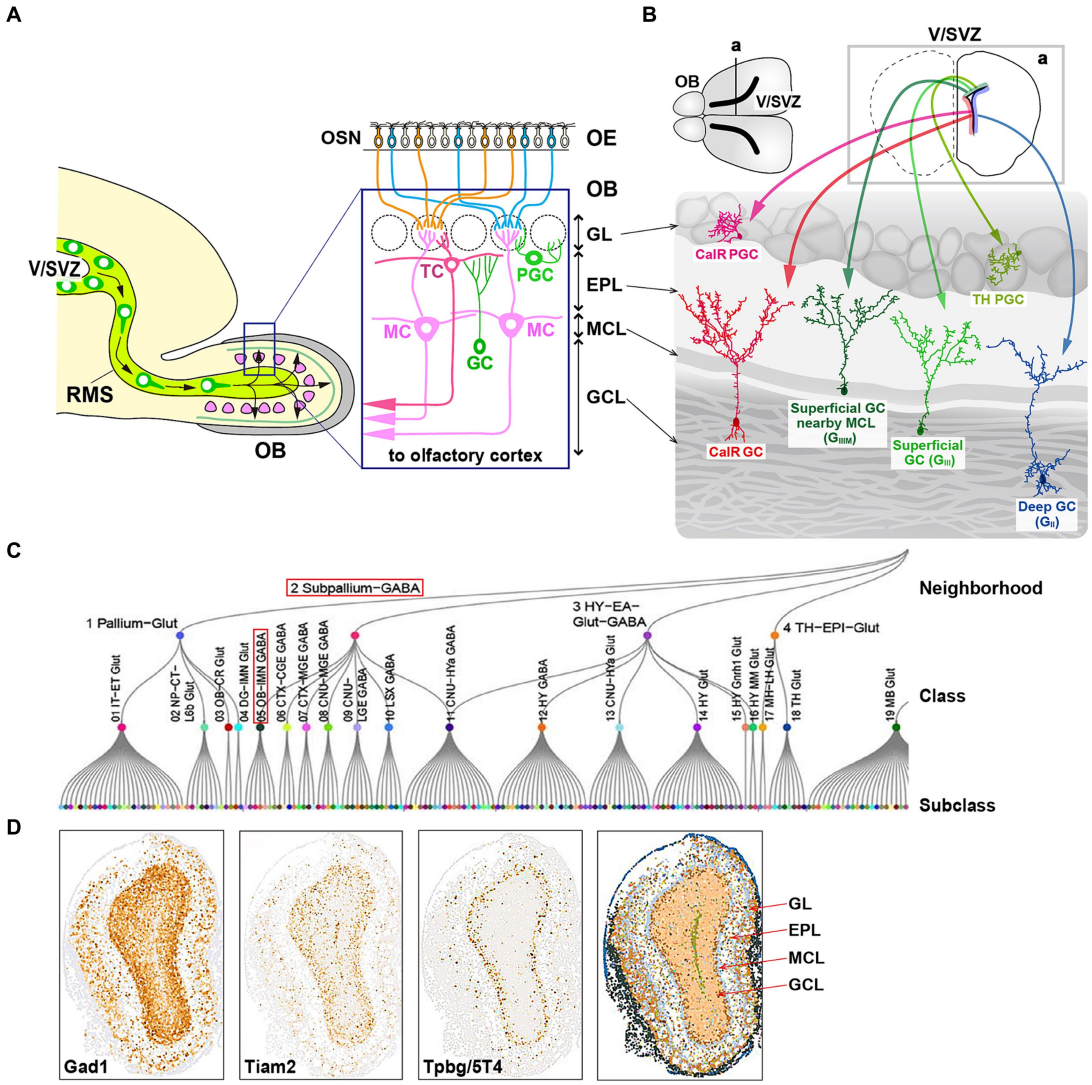
Cell fate of multiple subtypes of olfactory bulb (OB) interneurons. **(A)** The mammalian OB is structured into distinct layers: the glomerular layer (GL), external plexiform layer (EPL), mitral cell layer (MCL), and granule cell layer (GCL). Olfactory sensory signals from olfactory sensory neurons (OSN) in the olfactory epithelium (OE) are transmitted by excitatory projection neurons such as mitral cells (MCs) and tufted cells (TCs) to inhibitory interneurons like granule cells (GCs) and periglomerular cells (PGCs). **(B)** Distribution of neural stem cells in the ventricular-subventricular zone (V/SVZ) in specific areas. Adult OB interneurons are generated in different subregions of the V/SVZ (upper row; a), migrate through the rostral migratory stream (RMS), and subsequently differentiate into distinct subtypes of mature interneurons in the OB, including GCs (G_II_, G_III_, G_IIIM_, and CalR) and PGCs (TH and CalR). **(C)** Transcriptome cell type classification of whole mouse brain. A transcriptome classification tree is organized into 7 neighborhoods, 34 classes, and 338 subclasses. The subpallium-GABA neighborhood comprises 7 classes, one of which is the OB-IMN-GABA class containing 30 subclasses. Notably, the Frmd7 Gaba_1 subclass includes Tpbg/5T4 and Lgr6 genes. The content in panel **(C)** is adapted from the extended data figure in the study by Yao et al. (2023), with permission from the journal. **(D)** Spatial expression of the OBINH-3-[Gad1_Tpbg_Tiam2] subcluster genes in the mouse OB, based on the “Spatial-Portal” website (https://www.spatial-atlas.net/Brain/spatial.html). This site presents a spatial molecular atlas of the adult mouse central nervous system generated by Xiao Wang’s lab with STARmap PLUS, an imaging-based targeted *in situ* sequencing platform (Shi et al., 2023).

Sensory inputs are essential for the development and plastic modification of neural circuits in vertebrates (Lepousez et al., 2013). Olfactory sensory neurons (OSNs) detect individual odorants by expressing corresponding odorant receptors in OSNs on the olfactory epithelium (Mori and Sakano, 2011, 2021). The convergence of OSN axons on specific glomeruli within the olfactory bulb (OB) enables the activation of distinct neuronal circuits and facilitates the dendritic development of specific types of inhibitory interneurons through excitatory projection neurons in the OB (Mori and Sakano, 2011, 2021; Lepousez et al., 2013). Neural progenitors like transit-amplifying cells and neuroblasts are generated from neural stem cells (NSCs) in the ventricular-subventricular zone (V/SVZ) near the lateral ventricles, not only during early development but also in adulthood (Obernier and Alvarez-Buylla, 2019). These neuroblasts migrate through the rostral migratory stream (RMS) to the OB, where they mature into inhibitory interneurons that release gamma-aminobutyric acid (GABA), including granule cells (GCs) and periglomerular cells (PGCs) (Lledo and Valley, 2016) (Figure 1A). Within the OB, GCs and PGCs establish reciprocal connections with glutamatergic excitatory projection neurons like mitral cells (MCs) and tufted cells (TCs) (Figure 1A). GCs and PGCs receive glutamatergic inputs from MC/TC dendrites and return GABAergic outputs to them (Lledo and Valley, 2016). The survival and integration of newly born OB interneurons into existing neural networks are affected by odor-evoked neural activity (Lledo and Valley, 2016). Furthermore, olfactory deprivation and odor-rich environments, respectively, hinder and enhance the dendritic branching and spine formation of newborn OB interneurons (Bressan and Saghatelyan, 2021). Despite advancements in this area, the specific role of a particular subtype of newborn interneurons in modulating olfactory behaviors remains unclear due to the absence of distinct markers for them.

## 2 Embryonic and adult neurogenesis

The generation of OB interneurons in the rodent brain occurs in the V/SVZ during both embryonic and adult stages. Neurogenesis in the embryonic phase commences with neuroepithelial cells localized in the V/SVZ, which undergo a transformation into radial glial cells (RGCs) (Obernier and Alvarez-Buylla, 2019). This transition involves the loss of certain epithelial characteristics by neuroepithelial cells, such as tight junctions, and the acquisition of astroglial features marked by the expression of various astrocytic markers. Multiple intrinsic signals work in concert to facilitate this shift and ensure neurogenesis during embryonic development (Obernier and Alvarez-Buylla, 2019). Initially, RGCs function as fate-restricted neural progenitors, giving rise to transit-amplifying precursors or neuroblasts through symmetric mitosis, which further differentiate into neurons (Merkle and Alvarez-Buylla, 2006). In later stages of development, RGCs also generate glial cells like astrocytes and oligodendrocytes (Merkle and Alvarez-Buylla, 2006).

During the adult stage in the V/SVZ, radial glial-like NSCs generate various types of newborn interneurons that migrate to the OB through the RMS (Obernier and Alvarez-Buylla, 2019). The location of adult NSCs within areas of the V/SVZ determines the types of OB interneurons produced (Merkle et al., 2014) (Figure 1B). While it has been observed that adult NSCs originate from embryonic progenitors in the V/SVZ (Fuentealba et al., 2015; Furutachi et al., 2015), the timing of spatial determination of cell fate remains unclear, specifically whether neural progenitors in the V/SVZ subareas, where adult NSCs are present, are the same as those in the forebrain subareas where embryonic NSCs are. Adult neural progenitors are generated between embryonic days 13.5 and 15.5 but remain mostly inactive until reactivated postnatally. The majority of RGCs become active from late embryonic stages to postnatal day 15, with a small subset of NSCs remaining quiescent during embryonic development. These dormant NSCs are responsible for adult neurogenesis in the V/SVZ (Fuentealba et al., 2015; Furutachi et al., 2015). NSCs are activated to produce intermediate progenitor cells, which then give rise to neuroblasts. These neuroblasts, along with their immature neural precursors, migrate in chains through the RMS to the OB, where they differentiate into mature OB interneurons, including GCs and PGCs (Kaneko et al., 2017). In adult rodents, the RMS serves as a conduit for a substantial number of neuroblasts to reach the OB (Lledo and Valley, 2016; Obernier and Alvarez-Buylla, 2019). In an experiment involving adult mice (at postnatal weeks 8) labeled with bromodeoxyuridine (BrdU), over 20,000 newborn neurons were observed to reach the OB 14 days after BrdU injection, indicating robust neurogenesis and extensive plasticity in the V/SVZ (Yamaguchi and Mori, 2005). In addition, local neuronal proliferation in the OB was noted to be particularly active in the initial days following birth (Lemasson et al., 2005).

## 3 Fate map of Tpbg/5T4 GCs

Inhibitory GCs, characterized by their small cell bodies lacking axons, have spinous basal dendrites and a spinous apical dendrite that extends from the GC layer into the external plexiform layer (EPL) and contacts MC/TC lateral dendrites via large spines housing the reciprocal synapses (Shepherd et al., 2007) (Figure 1A). GCs, the predominant neuronal subpopulation within the OB, are involved in both recurrent and lateral inhibition of MCs/TCs at the EPL, where GCs receive glutamatergic synaptic input from MC/TC lateral dendrites and provide recurrent GABAergic synaptic output to them (Egger and Thomas Kuner, 2021). The vast majority (>95%) of neurons generated in the V/SVZ during adulthood differentiate into GCs within the OB (Winner et al., 2002). Initially, GCs are categorized into multiple subtypes based on the morphology of their dendrites and the positioning of their cell bodies within the GC layer. Studies utilizing horseradish peroxidase injection and Golgi staining have revealed that rodent GCs can be classified into three primary subtypes: intermediate (G_I_), deep (G_II_), and superficial (G_III_) subtypes (Shepherd et al., 2007), and further subcategorized into deep branching (G_IV_) and shrub branching (G_V_) subtypes (Merkle et al., 2007). Subsequently, mature GCs are identified by specific marker genes such as calretinin (CalR), Ca^2+^/calmodulin-dependent protein kinase II α (CaMKIIα), oncofetal trophoblast glycoprotein (Tpbg, also known as 5T4), metabotropic glutamate receptor 2 (mGluR2), and neurogranin (Imamura et al., 2006; Batista-Brito et al., 2008; Gribaudo et al., 2009; Merkle et al., 2014; Nagayama et al., 2014; Malvaut et al., 2017).

As mentioned above, adult NSCs in distinct subregions of the V/SVZ generate OB interneurons with diverse GC subtypes (Merkle et al., 2007, 2014) (Figure 1B). It was previously uncertain whether these spatial cell fate decisions are predetermined during early development. Recent advancements in gene barcoding techniques have enabled the mapping of single cell spatial distribution, revealing that different classes of adult-born OB interneurons are associated with specific types of embryonic-born neurons, depending on the V/SVZ subareas (Fuentealba et al., 2015) (Figure 1B). For instance, superficial GCs and dopaminergic (TH: tyrosine hydroxylase) PGCs originating in the dorsal V/SVZ during adulthood were found to be clonally linked to cortical neurons produced in the corresponding area during the embryonic development (Figure 1B). These findings suggest that the spatial determination of cell fate is established early during the embryonic stage in progenitor cells, which are shared NSCs between the embryonic forebrain and adult OB.

**Figure 2:**
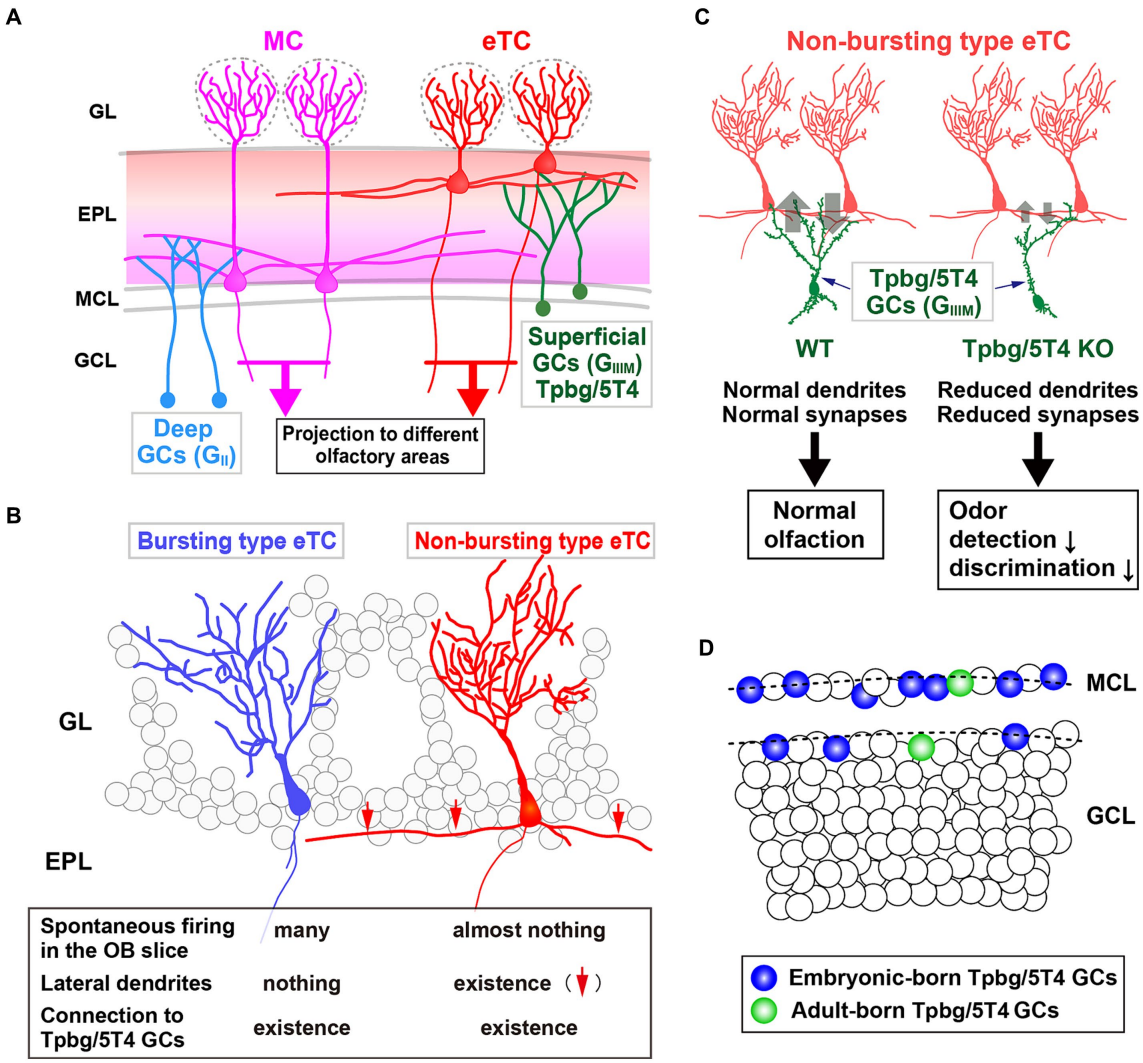
Role of the Tpbg/5T4 GC subtype within the OB neural circuit. **(A)** Schematic representation of the OB neural circuit. Superficial GCs nearby the MC layer (G_IIIM_) harboring Tpbg/5T4 GCs exhibits a preference for establishing connections with the lateral dendrites of external TCs (eTCs) located in the surface segment of the external plexiform layer (EPL). Conversely, GCs situated in the deep GC layer (G_II_) predominantly form connections with MCs in the deep segment of the EPL. These distinct pathways of MCs and TCs facilitate the transmission of varied odor information to discrete regions of the olfactory cortex. GL, glomerular layer; EPL, external plexiform layer; MCL, mitral cell layer; GCL, granule cell layer. **(B)** Two categories of eTCs. Tpbg/5T4 GCs establish dendritic synapses with two types of eTCs: burst type eTCs lacking lateral dendrites that exhibit frequent spontaneous firing; non-burst type eTCs possessing lateral dendrites that do not exhibit such firing behavior. **(C)** Tpbg/5T4 GCs connecting to non-burst type eTCs that display reduced dendritic branching in Tpbg/5T4-knockout (KO) mice. Notably, GABAergic inputs to non-burst type eTCs are significantly diminished in Tpbg/5T4 KO mice, whereas inputs to burst type eTCs remain unaffected. Consequently, olfactory functions such as odor detection and discrimination are impaired in Tpbg/5T4 KO mice. **(D)** Integration of Tpbg/5T4 GCs produced at embryonic and adult stages. Bromodeoxyuridine labeling reveals that embryonic-born Tpbg/5T4 GCs are predominantly integrated in the OB (depicted in blue), with minimal integration observed in adult-born Tpbg/5T4 GCs (depicted in green) (Takahashi et al., 2016).

Tpbg/5T4 GCs and CalR GCs are located in the MC layer and/or the superficial GC layer, respectively (Imamura et al., 2006; Nagayama et al., 2014) (Figure 1B). Tpbg/5T4 GCs are present in superficial GCs nearby the MC layer (G_IIIM_) based on their location and dendritic morphology (Imamura et al., 2006; Yoshihara et al., 2012; Fuentealba et al., 2015), while CalR GCs are in the other superficial GCs (G_III_) with a different cell lineage from Tpbg/5T4 (Merkle et al., 2014; Fuentealba et al., 2015; Yao et al., 2023) (Figure 1B). In addition, different subtypes of OB GCs were generated preferentially at different stages, e.g., from embryonic day 12.5 to postnatal day 30 (Batista-Brito et al., 2008). Tpbg/5T4 GCs, present in the superficial GC (G_IIIM_) layer, were produced mainly from embryonic day 15.5 to the day of birth (Sakamoto et al., 2014; Takahashi et al., 2016) (Figure 2D), whereas production of CalR GCs, present in the superficial GC (G_III_) layer, began immediately after birth and continued until postnatal day 56 (Batista-Brito et al., 2008). Tracing the genetic lineage of postnatally-born neurons has revealed that nearly 90% of GCs in the deep GC (G_II_) layer of mice at postnatal day 90 were marked as postnatally-born neurons, whereas only about 40 and 60% were labeled in the MC (G_IIIM_) layer and the superficial GC (G_III_) layer, respectively (Sakamoto et al., 2014). These results suggest that GCs generated at embryonic stages like Tpbg/5T4 are maintained in the superficial GC (G_IIIM_) layer (Figure 2D), while GCs produced after birth are mainly integrated into the deep GC (G_II_) layer.

High-throughput single-cell RNA sequencing (scRNA-seq) of the mouse OB albeit no spatial information identified 38 distinct cell clusters including 18 neuronal clusters, each of which has the similarity of transcriptional profiles and allows detailed classification of single-cell molecular markers (Tepe et al., 2018). Expression profiles with RNA *in situ* hybridization (ISH) from the Allen Brain Atlas (Lein et al., 2007) further classified the neuronal clusters into14 mature/immature inhibitory neuron clusters, one of which is the n12-GC-6 cluster consisting of Slc32a1 (vesicular GABA transporter), known to be expressed in mature inhibitory interneurons, Tpbg/5T4 and so on (Tepe et al., 2018). Recently, a high-resolution spatial transcriptomic atlas of cell types for the entire mouse brain was generated, in combination of a scRNA-seq dataset with a spatial transcriptomic dataset by multiplexed error-robust fluorescence ISH (MERFISH) (Chen et al., 2015; Yao et al., 2023). Through characterization of the molecular markers and regional specificity of each cell-type class, the subpallium-GABA class has been subdivided into seven subclasses, each likely originating from distinct developmental pathways (Figure 1C). Within these subclasses, one particular group comprises a combination of five non-neuronal cell types and four GC/immature neuron types, notably including the GABAergic OB immature neuron (OB-IMN-GABA) type (Yao et al., 2023) (Figure 1C). The OB-IMN-GABA type originates in the V/SVZ, migrates through the RMS, and matures into GCs and PGCs in the OB. During the developmental lineage of the OB-IMN-GABA type, neuroblasts in the SVZ and RMS express cell cycle-related markers like Top2a and Mki67. Upon exiting the RMS, immature OB interneurons exhibit markers such as Sox11 and S100a6, while mature OB interneurons are characterized by Frmd7 expression (Yao et al., 2023). The OB-IMN-GABA type is further categorized into 30 subtypes, one of which is the Frmd7 Gaba_1 subtype containing Tpbg/5T4 and Lgr6 (Leucine-rich repeat [LRR]-containing G-protein coupled receptor 6). Notably, a previous study by Yu et al. (2017) has indicated the co-expression of Lgr6 with Tpbg/5T4 in GCs at the superficial GC (G_IIIM_) layer.

Similarly, using an *in situ* RNA-sequencing method on brain slices along with the high-throughput scRNA-seq analysis (Zeisel et al., 2018), a high-quality spatial transcriptomic atlas of cell types has been made across the adult mouse brain to identify 26 major cell clusters, including 13 neuronal clusters (Shi et al., 2023). They are further classified into 190 neuronal subclusters that contains seven OB inhibitory neuron (OBINH) subclusters, one of which is the OBINH-3-[Gad1_Tpbg_Tiam2] subcluster. Based on the “Spatial-Portal” website (https://www.spatial-atlas.net/Brain/spatial.html), expression of Gad1 (Glutamate decarboxylase 1; GCs, and PGCs), Tiam2 (T-cell lymphoma invasion and metastasis 2; neuroblasts, GCs, and PGCs) and Tpbg/5T4 appears to be overlapped in the superficial GC (G_IIIM_) layer (Yoshizawa et al., 2002; Shi et al., 2023) (Figure 1D). Members of the Frmd7 Gaba_1 subtype (Yao et al., 2023) is similar but, not identical to those of the OBINH-3-[Gad1_Tpbg_Tiam2] subcluster (Shi et al., 2023), because in the superficial GCs nearby the MC layer (G_IIIM_), contamination of glutamatergic MCs seems to be unavoidable. These results demonstrate that the OBINH-3-[Gad1_Tpbg_Tiam2] subcluster represents a cell lineage associated with Tpbg/5T4 in the OB.

## 4 Physiological roles of Tpbg/5T4 GCs

Imamura et al. (2006) hypothesized that a group of membrane proteins present in specific layers within the OB neural circuit would play a role in the establishment of dendritic synaptic connections specific to those layers. They identified specific subtypes of OB interneurons, Tpbg/5T4 GCs and PGCs, within the superficial GC (G_IIIM_) and glomerular layers, respectively (Figure 2A). Tpbg/5T4 is a member of the LRR membrane protein family, characterized by a N-terminal extracellular domain comprising seven LRRs, each consisting of 24 amino acids (Imamura et al., 2006; Yoshihara et al., 2012; Zhao et al., 2014). Notably, Tpbg/5T4, among membrane proteins with extracellular LRRs, exhibits high conservation not only in mice (King et al., 1999) and humans (Myers et al., 1994) but also in non-mammalian species such as Drosophila CG6959 (Özkan et al., 2013) and zebrafish Wnt-activated inhibitory factor 1 (WAIF1) (Kagermeier-Schenk et al., 2011). It has been revealed by unilaterally naris occluded mice that expression of Tpbg/5T4 in OB interneurons is reliant on neural activity triggered by odors (Yoshihara et al., 2012). Further, experimental manipulations involving Tpbg/5T4 loss and gain of function have revealed that Tpbg/5T4 is essential for the dendritic branching of Tpbg/5T4 GCs in response to odor stimuli (Yoshihara et al., 2012). These findings suggest a crucial role for Tpbg/5T4 GCs in the processing of olfactory information within the neural circuits of the OB.

In the OB, inhibitory GCs synapse with excitatory projection neurons, such as MCs and TCs (Figure 2A). Previous studies have shown that early-born superficial GCs and late-born deep GCs tend to preferentially connect with the lateral dendrites of TCs and MCs, respectively (Geramita et al., 2016). In addition, Takahashi et al. (2016) identified two subtypes of external TCs (eTCs): non-burst type eTCs with inactive lateral dendrites; burst type eTCs that frequently exhibit spontaneous firing (Ma and Lowe, 2010) (Figure 2B). The apical dendrites of Tpbg/5T4 GCs establish GABAergic synapses with both non-burst and burst type eTCs. It has been shown utilizing OB slices from Tpbg/5T4 knockout (KO) mice that electrode stimulation induced GABA_A_ receptor-mediated postsynaptic currents in burst type eTCs, while significantly reduced responses were observed in non-burst type eTCs (Takahashi et al., 2016) (Figure 2B). Given the reciprocal dendritic synapses formed between OB GCs and projection neurons (Shepherd et al., 2007), the experiment exploring excitatory inputs from eTCs to Tpbg/5T4 GCs in Tpbg/5T4 KO mice revealed a notable decrease in those in Tpbg/5T4-KO than wild-type mice (Takahashi et al., 2016). This is consistent with reduction of dendritic branching in Tpbg/5T4-deficient cells (Yoshihara et al., 2012) (Figure 2C). These results demonstrate that neural activity in the non-bursting type eTCs is regulated by Tpbg/5T4 GCs (Figure 2B).

Would the reduced inhibition of non-burst type eTCs and decreased excitation of Tpbg/5T4-deficient GCs impact on olfactory behavior in mice? In order to assess the physiological significance of Tpbg/5T4 GCs in odor processing within the OB neural circuit, Takahashi et al. (2016) examined odor detection thresholds in wild-type and Tpbg/5T4 KO mice using the habituation-dishabituation test. The results revealed that Tpbg/5T4 KO mice exhibited significantly lower sensitivity to odor detection compared to wild-type mice, with a difference of approximately 100-fold. Furthermore, in an odor discrimination learning task, Tpbg/5T4 KO mice were unable to differentiate between two simultaneously presented odors but showed no impairment when the odors were presented individually (Takahashi et al., 2016). Notably, when exposed to a non-food-related odorant, Tpbg/5T4 KO mice displayed prolonged search times for buried food pellets. Conversely, these mice did not exhibit any difficulties in locating buried food pellets in the absence of other odors. To confirm the phenotype of global Tpbg/5T4 KO mice, the OB-specific Tpbg/5T4 knockdown (KD) experiment was performed by injection of Tpbg/5T4-shRNAs-expressing lentiviral vectors into both the lateral ventricles and OBs of postnatal day 1 mice, giving rise to knockdown specific to Tpbg/5T4 GCs in the adult OB (Takahashi et al., 2016). The OB-specific Tpbg/5T4 KD showed the same defect as the global Tpbg/5T4 KO in the odor detection thresholds and olfactory behavior tests, further demonstrating that Tpbg/5T4 GCs in the OB are required for both odor detection and odor discrimination behaviors as illustrated in Figure 2C.

## 5 Perspectives

The findings from olfactory behavior tests conducted on Tpbg/5T4 KO mice (Takahashi et al., 2016) diverge from those documented in prior research: either the inhibition or activation of neural activity in adult-born OB interneurons, including GCs, did not yield significant effects on odor detection and basic odor discrimination (Abraham et al., 2010; Alonso et al., 2012; Sakamoto et al., 2014). This discrepancy could be attributed to variations in the subtypes of OB interneurons manipulated genetically in each study. Given that OB interneurons can be categorized into multiple subtypes based on their expression markers, such as the OBINH-3-[Gad1_Tpbg_Tiam2] (Shi et al., 2023), it is postulated that each interneuron subtype forms a unique local circuit within the OB (Shepherd et al., 2007).

Sakamoto et al. (2014) proposed that around 25% of GCs in adult mice originate from embryonic NSCs, while Lemasson et al. (2005) indicated that postnatal neurogenesis peaks at postnatal day 7 and then decreases to one-third by postnatal day 60. The genetic trace experiment indicated that nearly 90% of GCs in the deep GC (G_II_) layer are produced continuously by postnatal day 90 after birth (Sakamoto et al., 2014). Consequently, adult-born GCs engineered to express the diphtheria toxin fragment A gene are predominantly integrated into the deep GC (G_II_) layer and tend to connect with MCs rather than TCs (Bardy et al., 2010), resulting in no impairment in odor detection and basic olfactory discrimination but a deficiency in complex learned olfactory discrimination (Sakamoto et al., 2011, 2014). Similarly, genetic inhibition and ablation of other OB GC subtypes, CaMKIIα GCs and neurogranin GCs, respectively, distributed across the GC layer, exhibits a necessity for learned olfactory discrimination (Malvaut et al., 2017; Gribaudo et al., 2021). Furthermore, chemogenetical inhibition of CalR GCs, by injection of the AAV harboring Gi-coupled DREADDs into the GC layer of CalR-Cre knockin mice, showed the impairment in complex olfactory discrimination, but not in simple learned olfactory discrimination (Hardy et al., 2018). This may be because CalR GCs are produced and integrated into the neural circuit during not only the neonatal stage but also the adult stage, even though they exist in the superficial GC (G_III_) layer (Batista-Brito et al., 2008).

In contrast, the genetic trace experiment indicated that GCs born during the embryonic stage appear to be retained in the MC and superficial GC layers (approximately 40 and 60% of GCs, respectively) in postnatal day 90 mice (Sakamoto et al., 2014). Consequently, Tpbg/5T4 GCs, generated during embryonic and perinatal periods and situated in the superficial GC (G_IIIM_) layer, demonstrate a requirement for odor detection and basic olfactory discrimination (Takahashi et al., 2016) (Figure 2C). Likewise, the GC-specific KO mice of GABA_A_ receptor β3 subunit (Gabrb3), where the AAV harboring Cre was injected into the GC layer of Gabrb3-floxed mice, showed reduced GABA_A_R-mediated inhibitory postsynaptic currents in GCs and increased recurrent inhibition in MCs. The effect on neural activity was restricted to part of the embryonic- and postnatal-born GCs, leading to impairment in discrimination both dissimilar and highly similar odors, but not in the learning of odor discrimination (Nunes and Kuner, 2015).

Recent studies have suggested that within the EPL of the OB, local dendrodendritic circuits may differentiate into parallel pathways for MCs and TCs, particularly eTCs (Fourcaud-Trocmé et al., 2014). The eTCs projecting to the anterior olfactory nucleus and the rostral part of olfactory tubercle (Igarashi et al., 2012), appear to be specialized in rapid odor detection and quick behavioral responses necessary for distinguishing between distinct odors. It is also possible that MCs projecting broadly to the olfactory cortex (Miyamichi et al., 2011; Igarashi et al., 2012) may excel in discrimination learning between closely related odors, such as enantiomers. I hypothesize that embryonic-born GCs, including Tpbg/5T4 GCs, are involved in fundamental olfactory responses crucial for survival, while adult-born GCs are more associated with learned olfactory behaviors (Alonso et al., 2012; Sakamoto et al., 2014). The distinct roles of embryonic- and postnatal-born GCs in olfactory processing and behavior may be attributed to the generation of different GC subtypes at various stages and subregions of the V/SVZ (Lemasson et al., 2005; Batista-Brito et al., 2008; Sakamoto et al., 2014; Fuentealba et al., 2015).

Interestingly, mammalian retina has a distinct laminar structure consisting of photoreceptor (rod and cone) cells, interneurons such as horizontal cells, bipolar cells (BCs) and amacrine cells (ACs), and retinal ganglion cells. Single-cell transcriptomics has revealed that Tpbg/5T4 belongs to a cluster of BCs divided into 15 clusters, and two clusters of ACs divided into 63 clusters within the mouse retina (Shekhar et al., 2016; Yan et al., 2020). Moreover, using scRNA-seq along with ISH images from the Allen Brain Atlas (Lein et al., 2007), it has been found that the subiculum pyramidal cells can be resolved into eight subclasses, one of which is the Tpbg/5T4 subclass (Cembrowski et al., 2018). These subclasses are mapped onto adjacent spatial domains, ultimately creating a complex layered and columnar organization with heterogeneity across the dorsal-ventral, proximal-distal, and superficial-deep axes in the mouse subiculum. Since OB neural circuits possess a notable high proportion of interneurons, glomerular columnar organizations, and intensive dendrodendritic communications, these observations suggest that Tpbg/5T4 plays a crucial role in shaping the laminar structure and function in the OB, retina, subiculum, and other brain regions.

## Author contributions

AT: Writing – original draft, Writing – review & editing.
